# Stabilization of metastable structures from high pressure or high temperature to ambient conditions

**DOI:** 10.1093/nsr/nwz072

**Published:** 2019-06-11

**Authors:** Zujin Shi

**Affiliations:** College of Chemistry and Molecular Engineering, Peking University, China

There are many kinds of metastable structures found in the phase diagrams of most materials. Although some structures have been found under extreme temperature and pressure, there are still structures not yet predicted in the existing phase diagram. Many kinds of silver iodide structures have been found under high pressure or high temperature including the α phase, rock-salt phase and potassium hydroxide-like structures [1]. However, it remains a big challenge to experimentally detect unpredicted structures and to stabilize these metastable structures of silver iodides.

**Figure 1:**
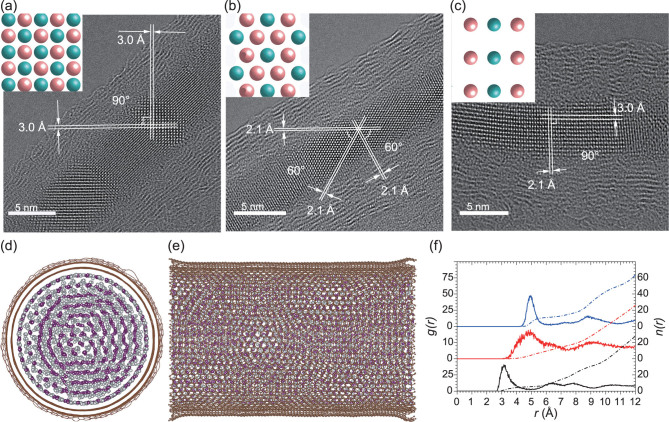
High-resolution transmission electron microscopy (HRTEM) images (a–c), molecular dynamics simulation (d, e) and radial distribution calculation results (f) of silver iodides inside MWCNTs [2].

In an article published in *National Science Review* [2], Zhang's group from Xi’an Jiaotong University developed a method to stabilize the metastable structures to ambient conditions by utilizing the inner narrow space of carbon nanotubes, inspired by the size effect on the phase transition of silver iodide [3] and the special inner physical and chemical environments of carbon nanotubes [2]. In the inner space of carbon nanotubes, high-pressure conditions were sustained for the material reactions. The pressure is mainly determined by the carbon nanotube inner diameters and the matching of the nanotube walls with the encapsulated materials. As a result, two types of otherwise unstable silver iodide structures were sustained under ambient conditions in the experiments.

One is the rock-salt phase of silver iodides (stabilized) in multi-walled carbon nanotubes (MWCNTs) with inner diameters of about 4–8 nm; this has been verified both experimentally (Fig. 1a–c) and theoretically (Fig. 1d–f). This structure of the rock-salt phase, stable under a pressure of 300 MPa–11.3 GPa, has been sustained under ambient conditions.

The other is a helix structure of silver iodide in single-walled carbon nanotubes (SWCNTs) with inner diameters of about 1.4 nm. Three outer iodine radial arms and three inner silver radial arms construct this particular structure, instead of the previously predicted single-walled inorganic nanotubes [4]. This new structure, which has not been recorded on the phase diagram, was simulated as stable with the Vienna *Ab-initio* Simulation Package (VASP), and needs deep investigation into its properties and potential applications.

Overall, this research has developed a new method to synthesize and sustain metastable structures from extreme conditions to ambient conditions. In addition, it should reduce the difficulties of undertaking research into these special structures and materials.
